# Investigation of EEG Activity Compared with Mean Arterial Blood Pressure in Extremely Preterm Infants

**DOI:** 10.3389/fneur.2018.00087

**Published:** 2018-02-26

**Authors:** Sujith S. Pereira, Stephen T. Kempley, David F. Wertheim, Ajay K. Sinha, Joan K. Morris, Divyen K. Shah

**Affiliations:** ^1^Neonatal Unit, Royal London Hospital, Barts Health NHS Trust, London, United Kingdom; ^2^Centre for Genomics and Child Health, Blizard Institute, Barts and the London School of Medicine and Dentistry, Queen Mary University of London, London, United Kingdom; ^3^Faculty of Science, Engineering and Computing, Kingston University, Kingston upon Thames, United Kingdom; ^4^Centre for Environmental and Preventive Medicine, Wolfson Institute of Preventive Medicine, Barts and The London School of Medicine and Dentistry, Queen Mary University of London, London, United Kingdom; ^5^Centre for Neuroscience and Trauma, Blizard Institute, Barts and the London School of Medicine and Dentistry, Queen Mary University of London, London, United Kingdom

**Keywords:** EEG, electroencephalogram, EEG continuity, blood pressure, preterm infant

## Abstract

**Background:**

Cerebral electrical activity in extremely preterm infants is affected by various factors including blood gas and circulatory parameters.

**Objective:**

To investigate whether continuously measured invasive mean arterial blood pressure (BP) is associated with electroencephalographic (EEG) discontinuity in extremely preterm infants.

**Study design:**

This prospective observational study examined 51 newborn infants born <29 weeks gestation in the first 3 days after birth. A single channel of raw EEG was used to quantify discontinuity. Mean BP was acquired using continuous invasive measurement and Doppler ultrasound was used to measure left ventricular output (LVO) and common carotid artery blood flow (CCAF).

**Results:**

Median gestation and birthweight were 25.6 weeks and 760 g, respectively. Mean discontinuity reduced significantly between days 1 and 3. EEG discontinuity was significantly related to gestation, pH and BP. LVO and CCAF were not associated with EEG discontinuity.

**Conclusion:**

Continuously measured invasive mean arterial BP was found to have a negative relationship with EEG discontinuity; increasing BP was associated with lower EEG discontinuity. This did not appear to be mediated by surrogates of systemic or cerebral blood flow. Infants receiving inotropic support had significantly increased EEG discontinuity on the first day after birth.

## Introduction

Infants born extremely preterm are at risk of cerebral injury (1) and the underlying mechanisms are poorly understood. Cerebral electrical activity in extremely preterm infants is influenced by multiple factors, for example administration of certain drugs and partial pressure of carbon dioxide in blood (PaCO_2_) can affect cerebral electrical activity in the days immediately after birth (2–4). Cerebral electrical activity has been previously examined in this group of infants in association with respiratory and metabolic indicators (5–7), circulatory measurements (3, 4, 8, 9), morphine (10–12), and inotropic therapy (13).

Circulatory parameters such as cerebral perfusion and blood pressure (BP) may affect cerebral electrical activity (3, 8, 9). It is still unclear as to whether mean BP could be associated with cerebral electrical activity in the extremely preterm. Studies have investigated the relationship between BP, left ventricular output (LVO), and cerebral electrical activity (8, 9, 14) but have been limited by patient numbers (9, 14) and the use of non-invasive BP measurements (8). There is a paucity of large prospective studies jointly examining the relationship between various clinical measurements including blood gas levels, the use of sedation and circulatory indicators with cerebral electrical activity in a recent cohort of extremely premature newborn infants.

We thus investigated mean arterial BP and EEG activity in the first 3 days in extremely preterm infants. In addition, we took account of pH, PaCO_2_, lactate, morphine administration, and a measure of cerebral perfusion.

## Materials and Methods

### The Study Population

Infants, both inborn and outborn, were eligible to participate if they were born at less than 29 weeks gestation, recruited within 12 h of age on the Neonatal Unit at the Royal London Hospital between February 2013 and April 2015. Formal exclusion criteria for this study included the presence of major congenital malformations and infants who did not have invasive arterial lines. This study received approval from the Research Ethics Committee (reference 12/LO/1553). Written parental consent was obtained prior to the start of the study.

### Amplitude-Integrated Electroencephalography (aEEG) Monitoring

Amplitude-integrated electroencephalography activity was recorded for 72 h in the majority of the infants using a 2-channel BRM3 monitor (BrainZ Instruments, Natus Medical Incorporated, ON, Canada) which provides a digital raw EEG signal output as well as the aEEG. After preparation of the scalp using NuPrep™ gel (Nuprep, D O Weaver & Co., Aurora, CO, USA) to reduce skin impedance, neonatal hydrogel electrodes (Neonatal Sensors, Natus Medical Incorporated, ON, Canada) were placed on the frontoparietal regions (C3-P3, C4-P4) bilaterally according to the international 10–20 system (15, 16). A 2-h artifact and seizure free electroencephalogram trace, confirmed by two observers (Sujith S. Pereira and Divyen K. Shah), recorded before and after measurement of the carotid artery blood flow was chosen for analysis.

### EEG Discontinuity

Single channel cross-cerebral (P3-P4) raw EEG data were exported to Microsoft Excel^®^ and continuity was analysed in 1-min epochs with software that we developed using MATLAB (The MathWorks, Inc., MA, USA) using a similar approach to that previously described (17). The system detected an interval if the absolute amplitude of the raw EEG was less than 20 µV with respect to the baseline for at least 6 s. The 20 µV threshold was chosen to reflect the fact that the EEG from preterm newborns is represented by more high voltage low frequency wave forms in contrast to full term newborns (18) and to help reduce any effects of background noise. The threshold level was thus chosen so as to reliably identify EEG bursts and distinguish them from background noise artefact in view of visual assessment of raw EEG characteristics. The 6 s criterion for defining an interval was chosen in order to exclude quiescent periods that are normally associated with tracé alternans. P3-P4 raw EEG was analysed, as C3P3 and C4P4 tend to be more susceptible to artifact due to the shorter inter-electrode distance. For each recording the mean of the total interval length per epoch, the discontinuity value, was calculated and expressed in seconds; this can also easily be expressed as a discontinuity proportion since the epoch length is constant.

### BP Monitoring

As invasive BP monitoring is considered the gold standard, only infants with invasive arterial lines were included in this study. Umbilical arterial catheters (UAC) were inserted aiming for the tip of the catheter to be maintained between 6th and 10th thoracic vertebral levels. Following insertion of the UAC, patency of the line was maintained by continuous infusion of heparinised saline. GE Healthcare medical systems monitor (Carescape Monitor B850) were used to trace the heart rate, BP, oxygen saturations levels, and respiratory rate. BP calibration was performed with the transducer being held in the mid axillary line at the start of the study and every 24 h thereafter. The UAC was only used after ensuring that the line was free from air bubbles, it sampled and flushed well and produced a good arterial waveform tracing. If the UAC was malpositioned or blocked, a peripheral arterial line was inserted and used after the above-mentioned precautions were taken. The heart rate, systolic, diastolic, and mean BP were monitored and downloaded every 10 s for the first week. A 2-h artefact free period of BP data, before measurement of common carotid artery blood flow (CCAF), was chosen for analysis.

### CCAF Measurement

Doppler ultrasound with a 7–15 MHz linear array probe (L15-7io Broadband compact linear array probe, Philips iE33, Bothwell, WA, USA) was used to measure the right CCAF volume on days 1 and 3. CCAF was used as a marker of cerebral blood flow using previously established methods (19) that have indicated good repeatability and reproducibility. An average of 5 right common carotid artery diameter and velocity time integral measurements were taken to calculate the blood flow volumes performed by one rater (SSP) after training. The right common carotid artery was used as it is furthest away from the ductus arteriosus and is less likely to be influenced by a patent ductus arteriosus (PDA) compared with the left common carotid artery. Whilst performing this examination, the presence or absence of a PDA on color-flow Doppler was also recorded.

### LVO Measurement

Doppler ultrasound with a 4–12 MHz sector array probe (S12-4, cardiac ultrasound probe, Philips iE33, Bothwell, WA, USA) was used to measure the LVO immediately after measuring the CCAF using methods that have been well established (20) on days 1 and 3. This method of estimation of LVO has been found to have good correlation with that measured using phase contrast MRI (21).

Mean arterial BP (averaged over a 2-h epoch) was compared with EEG discontinuity over the same epoch. Prior to the start of the study, care was taken to ensure that, for every infant, time was synchronised accurately to the minute across all the equipment used in the study. The relationship between EEG discontinuity to LVO, CCAF, and BP could thus be explored.

### Inotropic Support

A written policy for initiation of inotropic therapy was available at the cotside. Typically, infants were given a 10 ml/kg bolus of 0.9% saline and were then commenced on a dopamine infusion as necessary. Further inotropic agents were chosen based on the results of functional echocardiography that was performed on all infants in this study.

### Blood Gas Parameters

Blood gas parameters such as pH, PaCO_2_ and lactate values were chosen from single measurements that were closest to the measurements of CCAF and LVO on days 1 and 3.

### Statistical Methods

Data were tested for consistency with a normal distribution. Skewed data underwent logarithmic transformation for analysis. Effects on EEG discontinuity were analysed using independent samples *t*-tests for categorical variables, and Pearson’s correlation for continuous variables. For factors showing significant effects on discontinuity in these analyses (*p* < 0.05), a mixed effects multiple regression analysis was performed to identify predictors of EEG discontinuity, retaining gestation rather than birthweight as a measure of maturity. All statistical analyses were performed using SPSS v22 (Chicago, IL, USA) and Stata Release 12 (StataCorp LLC, College Station, TX, USA).

## Results

### Patient Characteristics

**Figure 1:**
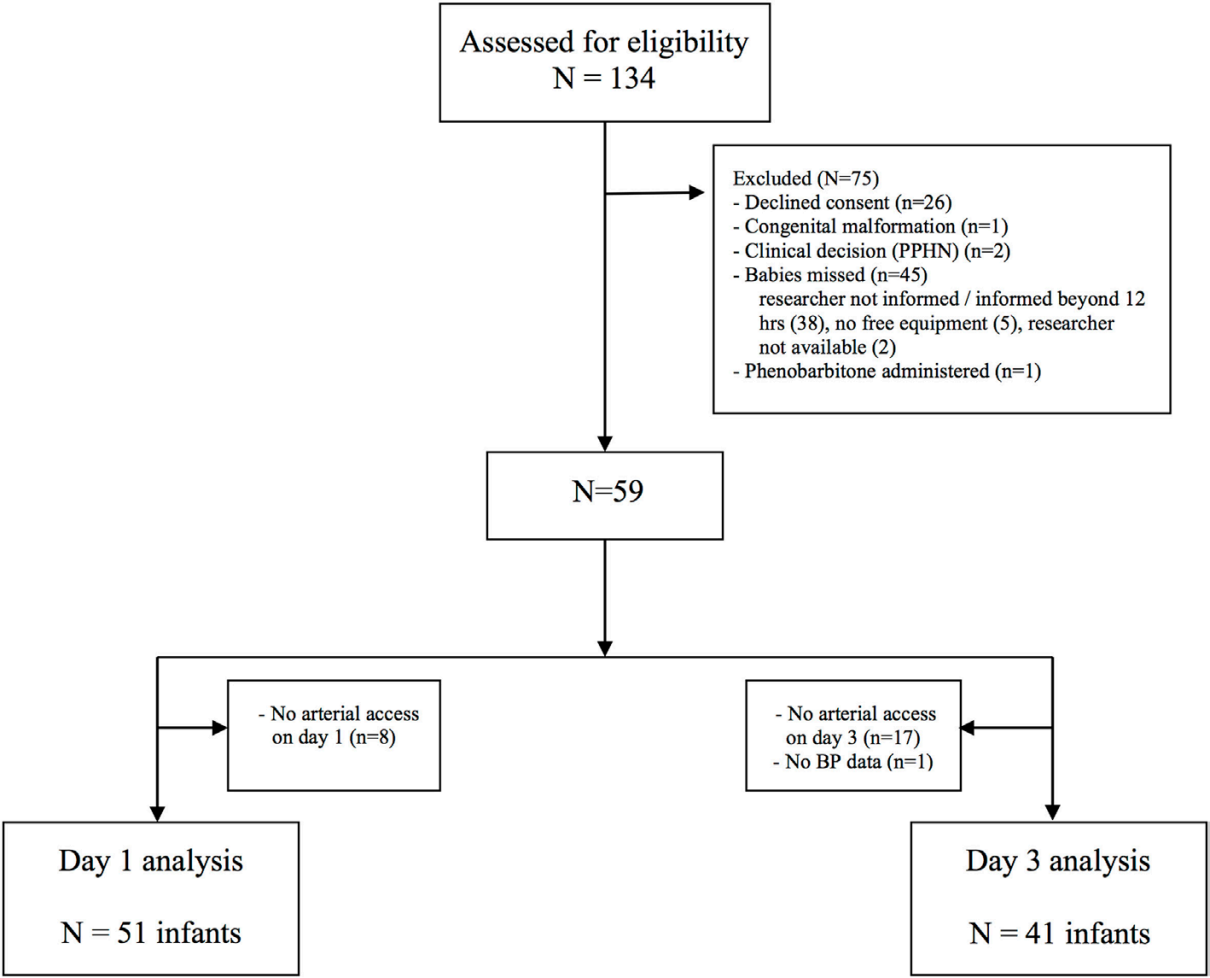
CONSORT diagram of recruitment of preterm newborns to the study.

Of 134 cases assessed for eligibility, 59 were recruited to the study (Figure 1). Fifty-one infants had invasive mean BP monitoring on day 1 and 41 infants on day 3. The clinical characteristics of recruited infants are shown in Table 1. The median [interquartile range (IQR)] age of days 1 and 3 scans were 18 (13–22) h and 74 (67–79) h, respectively. Sedation using morphine was administered in 16 infants on days 1 and 17 infants on day 3. One infant who received anticonvulsants in the first 72 h in view of suspected clinical seizures was excluded from analysis.

**Table 1:** Patient and clinical characteristics.

Patient characteristics	*n* = 51		
Gestational age (weeks)	25.6 (24.6–26.7)		
Birth weight (g)	760 (670–880)		
Males:females, *n* (%)	26 (51%):25 (49%)		
Mode of delivery, *n* (%)			
Vaginal delivery	45 (88%)		
Cesarean section	6 (12%)		
Cord gases^a^			
pH	7.28 (7.18–7.37)		
Base excess (mEq/l)	−5.5 (−9.3, −3.2)		
Apgar score			
1 min	5 (3–6)		
5 min	7 (6–9)		
Cranial USS findings in the first 72 h, *n* (%)			
Grade I or II	13 (25%)		
Grade III or IV	3 (6%)		
Death, *n* (%)			
<24 h	1 (2%)		
>24 h	7 (14%)		

**Clinical characteristics**	**Day 1 *n* = 51**	**Day 3 *n* = 41**	**Day 1 vs. 3 *p*-Value^b^**

Invasive ventilation, *n* (%)	49 (96%)	37 (90%)	0.26
PDA present, *n* (%)	46 (90%)	31 (74%)	0.06
Inotrope administered, *n* (%)	27 (53%)	14 (34%)	0.07
Morphine administered, *n* (%)	16 (31%)	17 (41%)	0.31
Presence of seizures, *n* (%)	7 (14%)	3 (7%)	0.33
pH	7.35 (7.30–7.41)	7.30 (7.27–7.35)	0.34
PaCO_2_ (mmHg)	36.0 (31.5–46.9)	41.2 (37.5–46.5)	0.47
Lactate (mg/dl)	19.8 (15.8–30.2)	17.1 (13.5–21.6)	0.002
Mean arterial BP (mmHg)	33 (29–36)	33 (30–36)	0.07
LVO (ml/kg/min)	167 (140–204)	217 (180–250)	<0.001
CCAF (ml/kg/min)	12 (9–14)	14 (12–18)	0.003
Analysable aEEG, *n* (%)	51 (100%)	36 (88%)	0.010
Mean discontinuity (s)	24 (16–31)	17 (9–25)	0.012

*Where not specified, all figures are expressed as median (interquartile range)*.

*SI conversion factors: to convert PaCO_2_ to mmol/L, multiply values by 0.133*.

*SI conversion factors: to convert lactate to mmol/L, multiply values by 0.111*.

*^a^n = 14*.

*^b^Paired t-test or chi-squared test*.

### EEG

**Figure 2:**
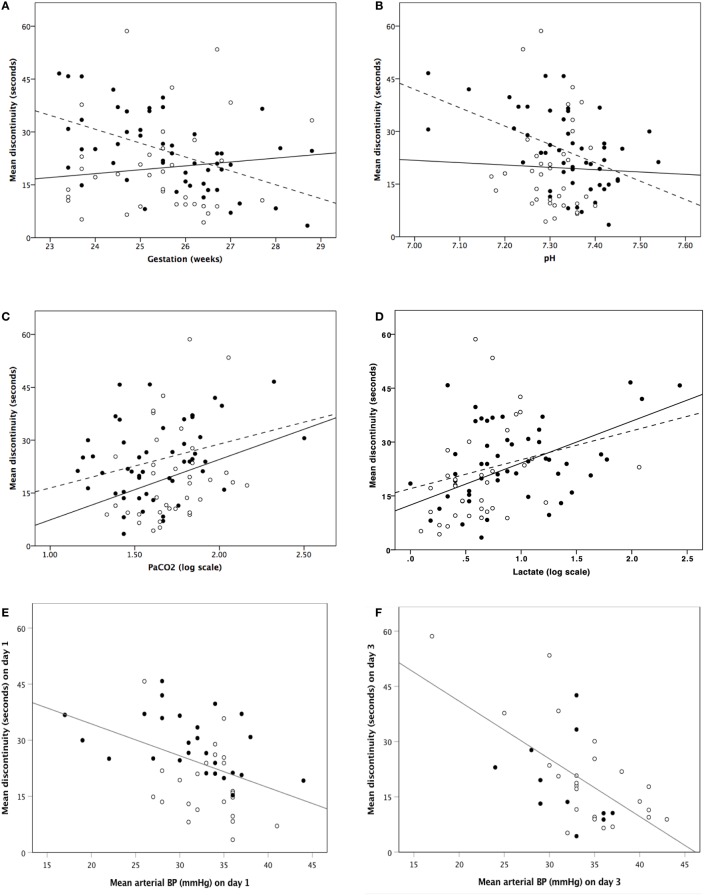
Correlation between mean discontinuity and gestation **(A)**, pH **(B)**, PaCO_2_ [log scale] **(C)**, lactate [log scale] **(D)**, and invasive mean blood pressure **(E,F)**. **(A–D)** Closed dots and dashed regression line represent day 1 and open dots and solid regression line represents day 3. **(E,F)** Closed dots represent infants who received inotropes and open dots represent infants who did not receive inotropes.

Analysable aEEG traces were obtained from 51 (100%) infants on days 1 and 36 (88%) infants on day 3. Infants with poor quality EEG signal and high impedance on day 3 were not included for analysis. From day 1 to day 3, EEG discontinuity decreased significantly (Table 1). EEG discontinuity decreased with increasing gestational age (GA) and this relation was more on day 1 than on day 3 (Figure 2A). Acidosis on day 1, higher PaCO_2_ and lactate, were related to lower voltage and a more discontinuous EEG (Figures 2B–D).

Morphine administration was significantly associated with increased mean discontinuity on both days. For those infants not receiving morphine, compared with those on morphine, median (IQR) mean discontinuity values were: 21 (13–25) vs. 36 (27–41) s (*p* < 0.001) on day 1 and 13 (9–21) vs. 23 (13–40) s (*p* = 0.022) on day 3.

Infants receiving inotropes had significantly (*p* < 0.001) suppressed mean discontinuity on day 1 only; on day 1 the proportion of infants studied who received inotrope treatment was 53%. For infants not receiving inotropes compared with infants on inotropes, median (IQR) mean discontinuity values were: 16 (12–24) vs. 29 (21–37) s. Inotrope administration did not appear to be associated with significant EEG discontinuity change on day 3 where a lower proportion (34%) of infants received inotropic support.

Continuously measured invasive mean arterial BP showed a significant relationship with EEG discontinuity; higher BP associated with lower EEG discontinuity on both day 1 and day 3 (Figures 2E,F). There was no correlation between LVO and CCAF and EEG discontinuity on day 1 or 3.

Using mixed effects multiple regression analysis (Table 2), we found that factors influencing mean EEG discontinuity include gestation (β = 3.57, *p* = 0.001), PaCO_2_ (β = 9.48, *p* = 0.009), lactate (β = 4.24, *p* = 0.028), morphine (β = 9.85, *p* < 0.001), and invasive mean arterial BP (β = −1.04, *p* < 0.001).

**Table 2:** Mixed effects multiple regression analysis between mean EEG discontinuity and clinical parameters.

Clinical parameters	*B coefficients*	SE	(95% CI)	*p*-Value
GA	3.57	1.04	(1.5, 5.6)	0.001
PaCO_2_	9.48	3.65	(2.3, 16.6)	0.009
Lactate	4.24	1.93	(0.5, 8.0)	0.028
Morphine	9.85	1.89	(6.1, 13.6)	<0.001
Mean arterial BP	−1.04	0.18	(−1.4, −0.7)	<0.001

## Discussion

The most unwell and immature infants would be expected to have the lowest EEG continuity; furthermore administration of inotropic support may be an indication of the degree to which the infant was unwell. However, additionally, this study found gestation, PaCO_2_, lactate, morphine administration and invasive mean arterial BP were significantly associated with EEG discontinuity in extremely preterm infants during the first 3 days after birth.

EEG discontinuity was related to GA in agreement with published data (22–26). We observed that acidosis and hypercapnia were associated with increased EEG discontinuity as previously reported (6, 7, 27–29). The suppression of EEG caused by hypercapnia may be exerted through changes in pH. Hypercapnia is associated with altered neuronal nuclear enzyme activity and a reduction in ATP and phosphocreatinine levels that reflect energy metabolism in animal models (30). As energy is required for maintenance of electrical activity in the brain (31), the resulting neuronal hyperpolarisation during hypercapnoea was associated with a reduction in the steepness, amplitude and duration of excitatory postsynaptic potentials (32).

In our study as in others (10, 12), morphine therapy was significantly associated with suppression of the EEG activity even though only 31% of infants on day 1 and 41% of infants on day 3 received morphine. The increase in EEG discontinuity noted with the administration of inotropes on day 1 may be due to prior hypotension triggering inotropic support, as there was no effect seen on day 3, by which time the BP levels would have stabilised; the proportion of infants receiving inotropic support fell from day 1 to day 3.

Mean invasive arterial BP was found to have a significantly negative relationship to EEG discontinuity on both day 1 and day 3 in this large cohort of infants. In contrast to other studies, our study was prospective with all infants having invasive BP monitoring, with continuous BP data being extracted every 10 s for the first week. West et al. (3) reported BP (non-invasive and invasive) data, acquired every minute, from 40 preterm infants at 12 and 24 h to be related to aEEG continuity at 12 and 24 h after birth. Infants in the lowest quartile for BP, which was below 31 mmHg, had lower aEEG continuity. EEG abnormalities are predictive of adverse long-term neurodevelopment in this group of infants (33). Victor et al. (9) showed EEG continuity to be normal in infants whose mean BP was above 30 mmHg. Our study has shown that increasing BP was associated with increased cerebral electrical activity.

There are several possible mechanisms by which EEG discontinuity could increase with lower BP levels, before reductions in cerebral perfusion affect cellular energy status. This could be postulated to be part of an intrinsic cerebral protective response to hypotension, with lower electrocortical activity reducing neuronal oxygen demand. In response to hypoxia–ischaemia, a neuroprotective adenosine mediated suppression of EEG has been reported in animal models (34, 35).

In our study, there was no consistent effect of blood flow parameters on EEG discontinuity that other studies have previously reported (8, 14). CCAF was not related to EEG discontinuity both on days 1 and 3. This would suggest that the relationship between systemic BP and EEG discontinuity is not simply mediated by alterations in systemic blood flow transmitted to the cerebral circulation. The median LVO measures were comparable to previously published data from hypotensive preterm neonates, but slightly lower than in more mature babies with higher BP and gestation (36).

Limitations of this study include that cerebral scanning and cardiac blood flow measurements were only carried out as a single measurement on one occasion on days 1 and 3 in the majority of infants.

## Conclusion

Our study suggests that continuously measured invasive mean arterial BP is negatively associated with EEG discontinuity and this does not appear to be mediated by systemic or cerebral blood flow parameters. Infants receiving inotropic support on the first day after birth had increased EEG discontinuity in comparison with those not receiving such support.

## Ethics Statement

This study was carried out in accordance with the recommendations of Research Ethics Committee (reference 12/LO/1553) with written informed consent from all subjects. All subjects gave written informed consent in accordance with the Declaration of Helsinki. The protocol was approved by the London-Surrey Borders Research Ethics Committee and NHS National Research Ethics Service.

## Author Contributions

SP: participated in conceptualising and designing the study, designed the data collection sheets, acquired and performed initial analysis of the data, drafted the initial manuscript, and approved the final manuscript as submitted. SK: conceptualised and designed the study, performed analysis of the data, reviewed and revised the manuscript, and approved the final manuscript as submitted. DW: designed the study, analysed the electroencephalograms, reviewed the manuscript, and approved the final manuscript as submitted. AS and DS: designed the study, performed analysis of the data, critically reviewed and revised the manuscript, and approved the final manuscript as submitted. JM: performed statistical analysis of the data, critically reviewed and revised the manuscript, and approved the final manuscript as submitted. All authors approved the final manuscript as submitted and agree to be accountable for all aspects of the work.

## Conflict of Interest Statement

David Wertheim is an inventor on a patent US5181520 “Method and apparatus for analysing an electro-encephalogram.” The other authors have no conflicts of interest to disclose.

## Abbreviations

aEEG, amplitude-integrated electroencephalography; BP, blood pressure; CCAF, common carotid artery blood flow; EEG, electroencephalography; GA, gestational age; IQR, interquartile range; LVO, left ventricular output; PaCO_2_, partial pressure of carbon dioxide in blood; PDA, patent ductus arteriosus; UAC, umbilical arterial catheter.
